# Safety, pharmacokinetics, and biologic activity of pateclizumab, a novel monoclonal antibody targeting lymphotoxin α: results of a phase I randomized, placebo-controlled trial

**DOI:** 10.1186/ar3554

**Published:** 2012-01-08

**Authors:** Brinda Emu, Diana Luca, Carolyn Offutt, Jane L Grogan, Bernadette Rojkovich, Marna B Williams, Meina T Tang, Jim Xiao, June H Lee, John C Davis

**Affiliations:** 1 Genentech, Inc, 1 DNA Way, South San Francisco, CA 94080, USA; 2 Polyclinic of the Hospital Brothers of St John of God in Budapest, 114 PO Box 54, Budapest H-1525, Hungary; 3 University of California at San Francisco, Box 0558, 185 Berry Street 5300, San Francisco, CA 94107, USA

## Abstract

**Introduction:**

Pateclizumab (MLTA3698A) is a humanized mAb against lymphotoxin α (LTα), a transiently expressed cytokine on activated B and T cells (Th1, Th17), which are implicated in rheumatoid arthritis (RA) pathogenesis. This study was conducted to assess the safety, tolerability, < NOTE: For clarity and per AMA/S-W Style, please restore the use of Oxford/serial commas (ie: David likes vanilla, strawberry, and chocolate ice cream) throughout. and biologic activity of single and multiple doses of intravenous (IV) or subcutaneous (SC) pateclizumab in RA patients.

**Methods:**

The single ascending dose (SAD) phase in patients with stable RA consisted of six cohorts (4:1 active:placebo at 0.3 mg/kg IV, 1.0 mg/kg IV, 1.0 mg/kg SC, 3.0 mg/kg IV, 3.0 mg/kg SC, and 5.0 mg/kg IV; *n *= 5/cohort). In the multiple ascending dose (MAD) phase, patients with prespecified RA disease activity received three doses of pateclizumab or placebo (4:1) every 2 weeks (1.0 mg/kg SC, *n *= 10; 3.0 mg/kg SC, *n *= 20; or 5.0 mg/kg IV, *n *= 5). Safety and tolerability were assessed throughout, and clinical activity was determined after three doses (Week 6).

**Results:**

We observed no serious adverse events (AEs) or dose-limiting toxicities, and the majority of AEs were mild to moderate. The pharmacokinetic profiles were linear, and clearance was independent of dose. Reductions in levels of serum CXCL13 were observed, supporting the biologic activity of pateclizumab on the LTα pathway. Patients receiving pateclizumab in the 3.0 mg/kg MAD group (3.0 mg/kg SC) demonstrated ACR20, ACR50, and ACR70 response rates at week 6 of 75%, 56% and 25%, respectively, compared with 57%, 29%, and 0% in the placebo group. The median Disease Activity Score in 28 joints, C-reactive protein, reduction was 28% for pateclizumab, versus 8.4% for placebo.

**Conclusions:**

Pateclizumabwas generally well-tolerated in RA patients. Preliminary evidence of clinical activity was observed in active RA patients at the dose level targeted for clinical effect.

## Introduction

Rheumatoid arthritis (RA) is a systemic autoimmune inflammatory disease associated with progressive joint damage, pain, fatigue, and disability. Despite advances in the treatment of RA, a significant proportion of patients do not achieve an adequate clinical response upon treatment with available therapies, and less than half of patients who do respond to therapy achieve complete remission [1]. Current biologic treatment options for the management of RA often target the proinflammatory cytokine TNF-α; however, these agents are associated with safety concerns, such as increased risk of serious infection [2]. In addition, intolerance to or contraindication of an existing therapy may further limit a patient's therapeutic alternatives.

Depletion of cellular subsets implicated in RA immunopathogenesis has demonstrated significant clinical efficacy [3]. Novel therapies that both target the cellular source of multiple proinflammatory cytokines and interrupt the autoimmune inflammatory cycle perpetuated in RA could lead to improved outcomes compared to existing treatments.

**Figure 1:**
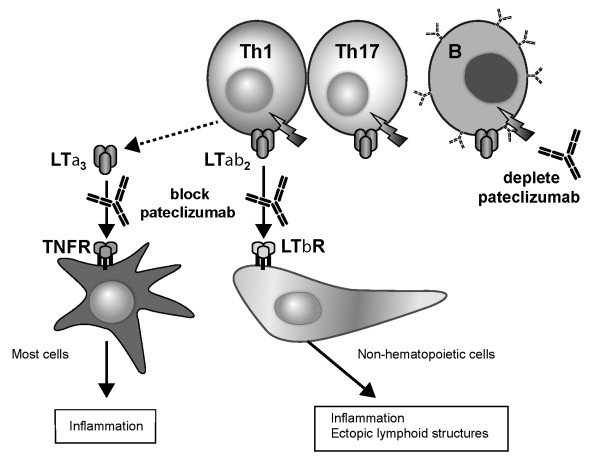
**Mechanism of action of MLTA3698A**. Lymphotoxin α (LTα) is a cytokine that is transiently expressed (as a secreted homotrimer or expressed together with LTβ on the cell surface) by subsets of activated T cells (Th1, Th17) and activated B cells (B) that are implicated in the pathogenesis of rheumatoid arthritis autoimmunity. Pateclizumabbinding to LTα expressed on the cell surface results in both the specific depletion of the activated cells and inhibition of immune cell trafficking and/or recruitment to inflammatory sites while leaving non-LTα-expressing cells such as Th2 unaffected. TNFR = tumor necrosis factor receptor; LTβR = lymphotoxin β receptor.

Lymphotoxin α (LTα), a member of the TNF superfamily, is both secreted (as the homotrimer LTα3) and transiently expressed on the cell surface of activated B, Th1 and Th17 cells, where it forms a complex with LTβ as LTα1β2 heterotrimers [4-6] (Figure 1). Soluble LTα3 binds TNF receptor (TNFR) types I and II, whereas cell-bound LTα1β2 heterotrimers bind LTβ receptors (LTβR), resulting in the downstream secretion of proinflammatory cytokines and chemokines, such as chemokine (C-X-C motif) ligand 13 (CXCL13) [7]. In addition, signaling through the LTβR pathway is required for the normal development of secondary lymph nodes and orchestration of robust germinal center architecture, and is also implicated in the development of ectopic lymphoid structures in chronically inflamed tissue [8].

LTα expression is also associated with the pathogenesis of RA. Ectopic lymphoid structures are present in synovial tissue from patients with RA [9], and both trimeric forms (LTα3 and LTα1β2) are elevated in the synovial fluid of patients with RA [10]. Furthermore, LTα, LTβ and LTβR transcripts are elevated in RA synovium [11-13].

A mouse-specific depleting mAb targeting surface LTα has been shown to ameliorate inflammation and arthritis in murine models of disease [6]. In the collagen-induced arthritis (CIA) model, mouse anti-LTα efficacy has been attributed to depletion of Th1 and Th17 cells, which are T-cell subsets that express LTα and are pathogenic drivers of disease. To date there are no therapies that specifically target these T-cell subsets.

Pateclizumab (MLTA3698A), a humanized mAb, specifically binds LTα in both the soluble LTα3 homotrimeric form and the surface-expressed LTα1β2 heterotrimer. Pateclizumab interferes with binding of LT trimers to its cognate receptors LTβR and TNFR and has the potential to deplete subsets of LTα-expressing cells. In this randomized, double-blind, placebo-controlled phase I study of 65 patients with active RA, we show the safety and activity of pateclizumab.

## Materials and methods

We designed this randomized, double-blind, placebo-controlled trial to evaluate the safety, tolerability, pharmacokinetics (PK), pharmacodynamics (PD), and preliminary evidence of biologic activity of pateclizumab in patients with RA. Study participants were recruited from 26 centers in the United States and Hungary. The institutional review boards at each study site approved the protocol, and all subjects provided their written informed consent. The trial was registered under US National Institutes of Health ClinicalTrials.gov identifier NCT00888745.

### Patients

Patients 18 to 75 years of age with a diagnosis of RA according to the American College of Rheumatology (ACR) 1987 revised criteria for RA [14] for at least 6 months were eligible for inclusion. Prior to randomization, patients must have been maintained on a stable disease-modifying antirheumatic drug (DMARD) regimen. Methotrexate up to 25 mg/week for 4 weeks, leflunomide up to 20 mg/day for 8 weeks, sulfasalazine up to 3 g/day for 6 weeks, hydroxychloroquine up to 400 mg/day for 8 weeks, or oral prednisone (or its equivalent) up to 10 mg/day at a stable dose for 4 weeks, were permitted.

Patients enrolled in the single ascending dose (SAD) stage had RA and were being treated with a stable DMARD regimen without prespecified disease activity. Patients enrolled in the multiple ascending dose (MAD) stage were being treated with a stable DMARD regimen, had received previous treatment with no more than one biologic agent and were also required to have moderate RA disease as defined by a swollen joint count (SJC) ≥ 5 (of 66), a tender joint count (TJC) ≥ 5 (of 68), and C-reactive protein (CRP) ≥ 1.0 mg/dl.

### Study design

In the SAD phase of the study, 30 patients (*n *= 5/cohort) were enrolled sequentially into six cohorts of five patients each (treatment allocation 4:1 pateclizumab:placebo) with four intravenous (IV) dose levels (0.3, 1.0, 3.0, and 5.0 mg/kg with 1-hour IV infusion; cohorts A, B, D and F, respectively) and two subcutaneous (SC) dose levels (1.0 and 3.0 mg/kg; cohorts C and E) (Table 1). Patients were enrolled into the next dose cohort 14 days after administration of the study drug from the last patient in the previous dose cohort. All patients in the SAD phase were followed for 12 weeks after the last dose of the study drug, with follow-up visits at Weeks 2, 4, 6, 8, and 12.

**Table 1:** Study schema for single-dose and multiple-dose escalation stages^a^

Stage dose and route	Patients, *n *(active:placebo)
SAD stage dose, route (cohort)	
0.3 IV (A)	4:1
1.0 IV (B)	4:1
1.0 SC (C)	4:1
3.0 IV (D)	4:1
3.0 SC (E)	4:1
5.0 IV (F)	4:1
MAD stage dose, route (cohort)	
1.0 SC (G)	8:2
3.0 SC (H)	16:4
5.0 IV (I)	4:1

^a^IV = intravenously; MAD = multiple ascending doses; SAD = single ascending dose; SC = subcutaneously.

In the MAD phase of the study, 35 patients were enrolled sequentially into three cohorts with the same treatment allocation of 4:1 active:placebo (1.0 mg/kg SC, *n *= 10; 3.0 mg/kg SC, *n *= 20; and 5.0 mg/kg 1-hour IV infusion, *n *= 5; cohorts G, H, and I, respectively). Prior to enrollment of the MAD dose cohorts, all available safety data were reviewed for all patients from the SAD phase of the study through at least 14 days of follow-up. Patients in the MAD cohorts received three doses of the study drug at 2-week intervals (0, 2, and 4 weeks). To enable detection of early clinical activity, 20 patients were enrolled in the target dose cohort 3.0 mg/kg SC (16 patients received active drug and 4 received placebo). All patients were followed for 12 weeks after the last dose of the study drug, with follow-up visits at Weeks 6, 8, 10, 12, and 16.

### Assessments

#### Safety

Safety data were collected at each visit and graded according to the National Cancer Institute Common Toxicity Criteria Adverse Event version 3.0 (National Cancer Institute, Frederick, MD, USA).

#### Pharmacokinetic assessments

In the SAD phase of the study, PK serum samples were obtained at predose, 1 hour (IV only) and 4 hours after dosing on study day 1, then on study Days 2, 4, 8, 15, 22, 29, 43, and 57. In the MAD phase of the study, PK serum samples were obtained on Day 1 (predose, 1 hour postdose IV only); Days 4, 8, and 15 (predose, 1 hour after the end of the IV infusion only); Days 18 and 29 (predose 1 and 4 hours after the end of the IV infusion only); and Days 30, 32, 36, 43, 57, 71, and 85. PK samples were analyzed using a validated ELISA with a lower limit of quantification of 100 ng/ml.

For anti-therapeutic antibody measurement, serum samples were collected at Day 1 (predose), 29 (predose), and 57 in the SAD stage. For the MAD stage, samples were collected at Day 1 (predose), 29 (predose), 57, and 85. On the basis of the assay validation data, the measured anti-pateclizumab responses are not likely to be enhanced by soluble LTα cross-reactivity, and the assay is formatted appropriately to reduce drug interference (data not shown).

#### Pharmacodynamic biomarker assessments

In the SAD phase of the study, PD serum samples were obtained at screening, Day 1 (predose) and Days 2, 4, 8, 15, 22, 29, 43, and 57. In the MAD phase of the study, serum samples were obtained at screening, Day 1 (predose) and Days 8, 15, 29, 36, 43, 57, 71, and 85. In all cohorts, levels of CXCL13 and total soluble LTα (free and drug bound LTα, which may include all trimeric forms) were assessed by ELISA with lower limits of quantification of 15.6 pg/ml and 100 pg/ml, respectively.

#### Clinical activity assessments

Exploratory analyses were conducted for the MAD stage to evaluate the effect of pateclizumab on the clinical response variables, including ACR20 and ACR50 (20% and 50% improvement, respectively, in tender or SJCs, as well as 20% and 50% improvement, respectively, in three of the other five American College of Rheumatology criteria for RA), Disease Activity Score in 28 joints (DAS28) responses and C-reactive protein (CRP) These analyses were performed at week 6, two weeks after the last dose of study drug.

### Statistical analysis

All patients who received at least one dose of the study drug were included in the safety, PK, and PD analyses. Adverse events (AEs), vital signs, laboratory tests, PK, and evidence of biologic activity were descriptively compared across the various treatment groups with no formal statistical testing.

For disease activity measurements, data were censored for patients who received a new or increased dose of a DMARD or who withdrew from the study. For all patients who received pateclizumab, censored data were imputed as the highest disease activity score in the corresponding cohort at the corresponding time point for active patients (or lowest activity for that patient, whichever was lower). For patients who received placebo, censored data were imputed as the last observation recorded for that patient.

PK was assessed by noncompartmental analysis using WinNonlin Professional version 5.2.1 software (Pharsight, Mountain View, CA, USA). Preliminary population PK modeling was conducted to estimate the bioavailability following SC dosing using all the available SAD and MAD PK data (NONMEM 7.2, ICON Development Solutions, Ellicott City, MD, USA).

## Results

### Study population

Of the 65 patients enrolled in the study, 45 patients were from the United States and 20 patients were from Hungary. Patient demographics were balanced between active drug and placebo recipients (Table 2) in both the SAD and the MAD stages of the study. As anticipated based on the inclusion criteria, patients in the MAD phase of the study had greater baseline disease activity than patients in the SAD phase. At baseline, patients in the MAD phase of the study had had a diagnosis of RA for a median of 8 years (IQR = 4 to 11, range = 0.6 to 30), with a median baseline CRP of 1.7 mg/dL (IQR = 1.3 to 3, range = 0.9 to 4.6), median erythrocyte sedimentation rate of 31 mm/hr (IQR = 24 to 64, range = 11 to 97), median SJC of 9 (IQR = 9 to 16, range = 5 to 45), median TJC of 17 (IQR = 11 to 31, range = 5 to 65) and a median DAS28-CRP score of 5.6 (IQR = 4.9 to 5.9, range = 3.9 to 7.9). In addition, in the MAD phase of the study, patients who received pateclizumab had higher baseline disease activity based on SJCs and TJCs, as well as higher CRP measurements.

**Table 2:** Baseline patient demographic and disease characteristics^a^

	SAD		MAD				
	
Demographics and disease characteristics	Placebo (*n *= 6)	**All **pateclizumab (*n *= 24)	Placebo (*n *= 7)	1.0 mg/kg SC (*n *= 8)	3.0 mg/kg SC (*n *= 16)	5.0 mg/kg IV (*n *= 4)	**All **pateclizumab (*n *= 28)
Demographics							
Sex, female:male	6:0	20:4	7:0	6:2	14:2	3:1	23:5
Median age, years (range)	57 (25 to 69)	57.5 (35 to 75)	57 (29 to 69)	56 (41 to 73)	56 (23 to 70)	54 (48 to 61)	57 (23 to 73)
Race, white:black	5:1	23:1	7:0	7:1	16:0	3:1	26:2
Region, USA:Hungary	All USA	All USA	3:4	6:2	5:11	2:2	13:15
Disease characteristics							
Median disease duration, years	3	5	5.4	9	7.5	7	8
RF- and anti-CCP-positive (%)	17	67	71.4	87.5	87.5	50.0	82.1
Concomitant medications (%)							
Prior anti-TNF-α	33	33	0	25	19	0	18
MTX	67	63	29	13	44	50	36
Leflunomide	0	4	43	38	6	25	18
Steroids	33	25	57	50	69	75	54
Median CRP, mg/dl (IQR)	0.2 (0.1 to 1.1)	0.7 (0.0 to 6.2)	1.6 (1.2 to 3.3)	2.1 (1.1 to 4.6)	1.6 (0.9 to 4.5)	3.7 (0.9 to 4.4)	2 (0.9 to 4.6)
Median ESR, mm/hour (IQR)	25.5 (12 to 48)	27 (2 to 59)	34 (24 to 65)	42.6 (14 to 86)	31 (11 to 97)	39.5 (22 to 66)	30.5 (11 to 97)
Median SJC (range)	12 (6 to 26)	14.5 (0 to 34)	6 (5 to 12)	13 (6 to 23)	7 (5 to 35)	12 (5 to 45)	10.5 (5 to 45)
Median TJC (range)	19 (12 to 38)	27.5 (0 to 66)	11 (5 to 33)	22 (5 to 60)	20 (7 to 65)	30 (12 to 56)	19.5 (5 to 65)
Median baseline DAS28-CRP (IQR)	4.8 (4.6 to 5.5)	5.4 (2.19 to 7.2)	5.7 (3.9 to 6.0)	6.0 (4.8 to 7.9)	5.4 (4.0 to 7.5)	5.7 (4.2 to 7.0)	5.6 (3.9 to 7.9)

^a^CCP = cyclic citrullinated peptide; CRP = C-reactive protein; DAS28-CRP = Disease Activity Score in 28 joints, C-reactive protein; ESR = erythrocyte sedimentation rate; IV = intravenously; MAD = multiple ascending dose; MTX = methotrexate; RA = rheumatoid arthritis; RF = rheumatoid factor; SAD = single ascending dose; SC = subcutaneously; SJC = swollen joint count; TJC = tender joint count.

### Patient disposition

Overall, 97% of patients in the pateclizumab-treated groups and 100% of patients in the placebo groups completed the study. One patient in cohort G (MAD 1 mg/kg SC) who received active drug discontinued the study at day 20 because of an RA flare after having received two doses of the study drug. Clinical activity data were censored for an additional seven patients in the MAD phase of the study (6 of 28 receiving pateclizumab and 1 of 7 receiving placebo) who received adjunctive steroid therapy during the course of the study.

### Safety

Pateclizumabwas well-tolerated during the study. There were no serious AEs and no dose-limiting toxicities. No AEs resulted in the withdrawal of the study drug. Twenty patients in the SAD phase reported a total of 44 AEs. The overall incidence of AEs was slightly higher in pateclizumab-treated patients than in placebo-treated patients (71% and 50%, respectively) (Table 3). Twenty patients in the MAD phase reported a total of 54 AEs, with both treatment groups experiencing comparable rates of AEs (57% in pateclizumab-treated patients and 57% in placebo-treated patients). Nearly all (97%) of the AEs were grade 1 or 2. All three grade 3 AEs in patients receiving pateclizumabwere reported as unrelated to study drug administration.

**Table 3:** Treatment-emergent adverse events by preferred term occurring in at least two patients from all pateclizumab-treated groups combined during either single or multiple ascending dose phases^a^

	SAD cohorts, *n *(%)		MAD cohorts, *n *(%)				
	
Adverse events	Placebo (*n *= 6)	All active (*n *= 24)	Placebo (*n *= 7)	1.0 mg/kg SC (*n *= 8)	3.0 mg/kg SC (*n *= 16)	5.0 mg/kg IV (*n *= 4)	All active (*n *= 28)
Any event	3 (50.0)	20 (66.7)	4 (57.1)	7 (87.5)	8 (50.0)	1 (25.0)	20 (57.1)
Fatigue	0 (0.0)	2 (8.3)	1 (14.3)	0 (0.0)	0 (0.0)	0 (0.0)	0 (0.0)
Rheumatoid arthritis	0 (0.0)	2 (8.3)	0 (0.0)	1 (12.5)	1 (6.3)	0 (0.0)	2 (7.1)
Rash	0 (0.0)	2 (8.3)	0 (0.0)	0 (0.0)	0 (0.0)	0 (0.0)	0 (0.0)
Diarrhea	0 (0.0)	1 (4.2)	0 (0.0)	3 (37.5)	1 (6.3)	0 (0.0)	4 (14.3)
Vomiting	0 (0.0)	0 (0.0)	0 (0.0)	2 (25.0)	0 (0.0)	0 (0.0)	2 (7.1)
Nasopharyngitis	0 (0.0)	0 (0.0)	0 (0.0)	0 (0.0)	2 (12.5)	0 (0.0)	2 (7.1)
Headache	0 (0.0)	1 (4.2)	0 (0.0)	2 (25.0)	1 (6.3)	1 (25.0)	4 (14.3)
Rhinorrhea	0 (0.0)	0 (0.0)	0 (0.0)	1 (12.5)	1 (6.3)	0 (0.0)	2 (7.1)

^a^IV = intravenously; MAD = multiple ascending dose; SAD = single ascending dose; SC = subcutaneously.

There was no trend toward an increased incidence of AEs with increasing doses of pateclizumab. The most common AEs reported in the active arm were musculoskeletal, gastrointestinal, and headache. There was a single injection site reaction in a patient who experienced injection site pain in the 3.0 mg/kg SC cohort in the SD stage of the study. There were no increased rates of infections in patients receiving pateclizumabcompared to placebo. In addition, there were no significant differences over time in total lymphocytes, T cells, or B cells between patients receiving pateclizumabcompared to placebo (data not shown).

### Immunogenicity

Of the 52 patients treated with pateclizumab, anti-pateclizumabantibodies were detected in a total of seven patients. In the IV cohorts, 1 of 20 patients was anti-pateclizumabantibody-positive. In the SC groups, 6 of 32 patients were anti-pateclizumabantibody-positive. There were no anti-pateclizumabantibodies detected in placebo-treated patients or at any predose time points.

### Efficacy outcomes

Clinical activity was assessed at Week 6, 2 weeks after the last dose of the study drug. Cohort H was used for evaluation of biologic activity based on the dose (3.0 mg/kg SC) and expanded sample size (*n *= 20). Baseline disease activity scores, as measured by DAS28-CRP and CRP, were comparable between those patients receiving pateclizumabin cohort H and patients receiving placebo (pooled placebo data across all MAD cohorts).

**Figure 2:**
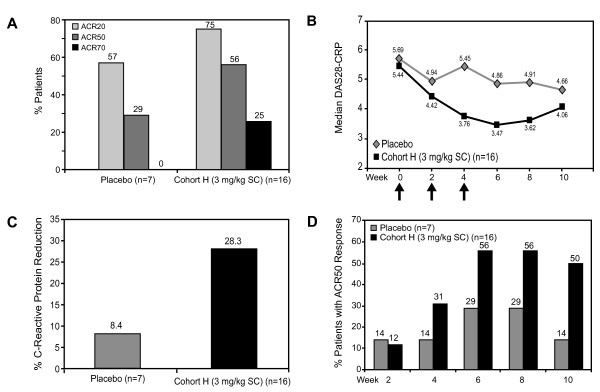
**Clinical activity in patients receiving 3 mg/kg SC pateclizumabor placebo**. ACR20, ACR50 and ACR70 = 20%, 50% and 70% improvement, respectively, in tender or swollen joint counts, as well as 20%, 50% and 70% improvement, respectively, in three of the other five American College of Rheumatology criteria for rheumatoid arthritis; DAS28-CRP = Disease Activity Score in 28 joints, C-reactive protein; SC = subcutaneously.

A total of 75%, 56%, and 25% of patients treated with 3 mg/kg pateclizumabSC achieved an ACR20, ACR50, and ACR70 responses, respectively (Figure 2A). In comparison, 57%, 29%, and 0% of the patients receiving placebo achieved ACR20, ACR50, and ACR70 scores. Patients receiving pateclizumaband placebo had median baseline DAS28-CRP scores of 5.44 and 5.69. At week 6, patients receiving pateclizumabhad a median DAS28-CRP score of 3.47 with a median reduction of 1.22. Placebo patients' median score was 4.86, with a median reduction of 0.82 (Figure 2B). At Week 6, median CRP measurements had decreased 28% from baseline in pateclizumab-treated patients compared to 8% among placebo-treated patients (Figure 2C). Patients receiving multiple doses pateclizumabat 3.0 mg/kg SC indicated preliminary evidence of clinical activity on all three prespecified end points. When patients were followed to Week 10 (6 weeks after the last dose of the study drug), ACR50 responses were sustained, with 50% of patients receiving pateclizumabmaintaining an ACR50 response compared with 14% of placebo-treated patients (Figure 2D).

Patients receiving pateclizumabin a lower-dose MAD cohort (cohort G, 1.0 mg/kg SC; *n *= 10) did not show evidence of clinical activity. Data from two patients were imputed (one patient received prednisone on day 2 of the study and another withdrew after two doses because of RA flare). Thus, at the 6-week end point, our sample size was too limited for characterization of efficacy. In the highest-dose cohort (cohort I, 5.0 mg/kg IV), the presence of clinical activity was observed in a single patient (one of four receiving pateclizumab) with an ACR70 score; however, the interpretation of a dose response is limited because of the small number of patients in this cohort.

In the expanded cohort (3.0 mg/kg SC), 7 of 16 patients were taking methotrexate and 3 of 16 patients had previously received TNF blockade. There was no clear difference in efficacy based on the use of methotrexate. Four of seven patients taking methotrexate achieved a score of ACR50, whereas three of nine patients not receiving methotrexate achieved an ACR50 score. The median reductions from baseline in DAS28-CRP scores were 1.37 and 1.07 (mean reductions in DAS28-CRP of 0.84 and 1.04,) in those who were and were not receiving methotrexate. Of the three patients who had previously received TNF blockade, none achieved an ACR50 score. Four patients in the 3.0 mg/kg SC expanded cohort developed anti-pateclizumabantibodies. Three of these patients also achieved an ACR50 score, despite the development of anti-pateclizumabantibodies.

### Pharmacokinetics

**Figure 3:**
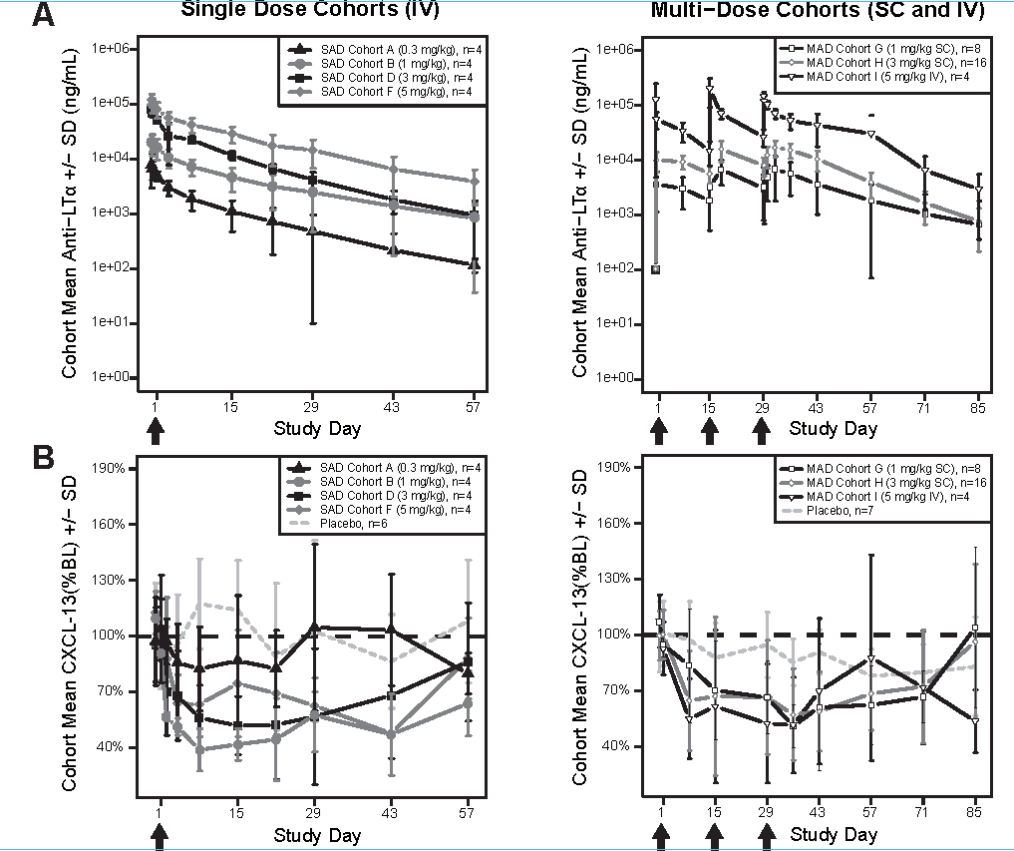
**Mean (± SD) serum pateclizumabpharmacokinetic profiles (A) and CXCL13 profiles (B) by cohort**. CXCL13 is presented as the percentage change from baseline. Arrows indicate pateclizumabdoses. BL = baseline; CXCL13 = chemokine (C-X-C motif) ligand 13; IV = intravenously; LTα = lymphotoxin α; MAD = multiple ascending dose; SAD = single ascending dose; SC = subcutaneously.

Pateclizumabconcentration and time profiles displayed biexponential decay after IV administration (Figure 3A). Overall, pateclizumabexposure, as measured by maximum serum concentration (C_max_) and area under the concentration time curve (AUC), were approximately dose-proportional after single or multiple doses. After a single dose, the cohort mean clearance (CL) and terminal half-life (t_1/2_) ranged from 4.54 to 7.03 ml/day/kg, and 8 to 15 days, respectively. The bioavailability following SC dosing was approximately 43%. After three doses of pateclizumabat 1 or 3 mg/kg SC, or 5 mg/kg IV at 2-week intervals (0, 2, and 4 weeks), the mean accumulation index ranged from 1.77 to 1.88. The mean CL and t_1/2 _values from the MAD cohorts were similar to those from the SAD cohorts.

### Pharmacodynamics

Decreases in CXCL13 after administration of pateclizumabwere observed in the SAD and MAD phases in all cohorts receiving doses ≥ 1 mg/kg pateclizumab (Figure 3B). Decreases in CXCL13 levels following pateclizumabadministration indicated biological activity. Increases in soluble LTα were observed in all cohorts given anti-LTα, with minimal elevation at the lowest doses (0.3 mg/kg IV and 1 mg/kg SC) and no changes in the placebo group. Maximal increases occurred at 3 mg/kg SC. These results are consistent with the expected formation of pateclizumab-LTα complexes and an increased half-life of soluble LTα upon binding to pateclizumab. Similar results were observed in preclinical studies in cynomolgus monkeys [15].

### Correlative analysis of pharmacodynamics and pharmacokinetics

Maximum decreases in CXCL13 (PD effects) were consistently observed at serum drug concentrations of ≥ 1 to 10 μg/ml across all anti-LTα cohorts with ≥ 1 mg/kg doses (Additional file 1). There was no apparent correlation between decreases in CXCL13 and pateclizumabC_max_, or AUC.

## Discussion

In this phase I study, pateclizumab, a humanized mAb that specifically binds LTα, was well-tolerated in patients with active RA. Using an expanded cohort approach, preliminary evidence of clinical activity was observed at the prespecified target dose of 3 mg/kg SC as measured by reduction in DAS28-CRP scores, reduction of CRP values, and achievement of ACR20, ACR50, and ACR70 responses. PK data revealed linear biphasic kinetics with dose proportionality. CXCL13 reduction as a PD marker was observed, supporting the anticipated effect of pateclizumabon downstream signaling via the LTα pathway.

Pateclizumabis a humanized mAb against the cytokine LTα. It is thought to act both by selectively depleting a specific subset of LTα-expressing activated T and B cells and by blocking LTα-mediated signaling (Figure 1). LTα is expressed on subsets of Th1 and Th17 T cells, as well as on activated B cells, but is absent from other subsets, such as Th2 [6]. Pateclizumablikely exerts its effects farther upstream than TNF inhibitors by targeting the cellular source of multiple proinflammatory cytokines. In a preclinical model of CIA, a murine antibody targeting LTα showed clinical efficacy by specifically depleting LTα-expressing lymphocytes, resulting in decreased joint pathology and rapid reduction of TNF-α, IL-1β and IL-6 levels within the hindpaw [6]. Importantly, the murine antibody had no effect in a Th2-driven model of asthma, demonstrating selectivity of anti-LTα for Th1 and Th17 cells.

This phase I study of patients with moderate to severe RA was designed to assess the safety and tolerability of pateclizumaband to evaluate preliminary evidence of clinical activity. pateclizumabwas well-tolerated in patients receiving IV or SC administration of either single or multiple doses. The majority of AEs were grade 1 or 2, with no serious AEs and no withdrawal of the study drug due to AEs. Importantly, no serious infections were reported during the study, and no increased rate of infections was reported in patients receiving pateclizumabcompared with placebo. PK analysis revealed that exposure to pateclizumabwas approximately dose-proportional. Anti-pateclizumabantibodies were detected in 7 of 52 pateclizumab-treated patients, but without any clinical sequelae.

CXCL13 is a chemokine induced downstream of LTβR signaling which has been implicated in the formation of ectopic lymphoid structures [11,12,16]. Elevated CXCL13 serum levels have been associated with synovitis in RA [17], and serum CXCL13 is decreased following treatment of RA patients with TNF-α inhibitors and anti-CD20 rituximab [17-19], thus implicating CXCL13 as a robust biomarker of RA disease activity. In our PD evaluations, pateclizumabadministration maximally decreased CXCL13 levels in all dose cohorts ≥ 1.0 mg/kg SC or IV. The reduction in CXCL13 most likely reflects an effect on downstream events following interruption of the LTα pathway.

The 3.0 mg/kg SC cohort in the MAD phase of the study consisted of an expanded cohort designed to evaluate clinical activity of pateclizumabat the target dose. Clinical response variables were assessed at week 6 (2 weeks after the last dose of drug). ACR20, ACR50, and ACR70 response rates were seen in 75%, 55%, and 25% of patients. In addition to ACR20, ACR50, and ACR70 responses achieved in this patient cohort, these patients showed substantial decreases in DAS28-CRP scores and CRP levels compared to placebo-treated subjects. Overall, for all measures evaluated, there was preliminary evidence of clinical activity in patients receiving pateclizumabcompared to placebo. Furthermore, evidence of sustained clinical activity was observed, with 50% of patients maintaining an ACR50 score up to 6 weeks after receiving their last dose of the study drug. The concomitant use of methotrexate did not affect clinical activity results. None of the three patients who had previously received TNF blockade achieved an ACR50 score. However, the small number of these patients limits the interpretation of activity in these subpopulations of patients who may be receiving (or may have received) other medications targeting RA disease.

The limitations of this study include the small sample size, which restricts interpretation of the study results. Regarding clinical activity, the other MAD dose cohorts (1.0 mg/kg SC and 5.0 mg/kg IV) were not designed as expanded cohorts, thus the ability to detect a dose response was limited by the small number of patients within these cohorts. In addition, patients were recruited from only two countries for this study (the United States and Hungary). Larger global studies will allow the evaluation of the effect of pateclizumabacross a broader RA patient population. Baminercept (a human LTβR-human immunoglobulin G1 (IgG1) fusion protein) failed to show clinical benefit in a phase II trial in patients with RA [20,21]. Although both pateclizumaband baminercept block LTαβ-induced signaling (via the LTβ receptor) [7], it is important to note that pateclizumabis distinct in its potential to selectively deplete LTα-expressing cells. In a preclinical murine model of established arthritis, a depleting murine anti-LTα antibody had efficacy comparable to that of anti-TNF-α antibodies, whereas an LTβR/Ig blocker had no effect [6]. In addition, this murine antibody demonstrated efficacy in mice that were refractory to anti-TNF administration [22]. Together these data suggest that the depleting anti-LTα mAb pateclizumabhas great potential for clinical activity in RA.

## Conclusions

Inhibition of the LTα pathway provides a unique approach by which to abrogate proinflammatory responses in RA pathology. By blocking signaling through the LTα pathway, as well as by depleting LTα-expressing cells, which are dominant sources of TNF-α and IL-6, pateclizumabis likely to work upstream of TNF-α blockade. This mechanism may provide an attractive alternative therapeutic approach for patients with moderate to severe RA. In this phase I study, pateclizumabwas well-tolerated and provided a linear PK profile when delivered subcutaneously. Given preliminary evidence of biologic and clinical activity, further evaluation of its clinical efficacy is warranted in larger clinical trials. To further characterize the safety, PK, PD and clinical activity of pateclizumab, a phase II study of RA patients without an adequate response to DMARDs is in progress.

## Abbreviations

ACR: American College of Rheumatology; AE: adverse event; anti-CCP: anti-cyclic citrullinated peptide; AUC: area under the concentration time curve; CIA: collagen-induced arthritis; CL: mean clearance; C_max_: maximum serum concentration; CRP: C-reactive protein; DAS28-CRP: Disease Activity Score in 28 joints, C-reactive protein; DMARD: disease-modifying antirheumatic drug; ELISA: enzyme-linked immunosorbent assay; IL: interleukin; IV: intravenous; LTα: lymphotoxin α; mAb: monoclonal antibody; MAD: multiple ascending dose; MTX: methotrexate; PD: pharmacodynamics; PK: pharmacokinetics; RA: rheumatoid arthritis; RF: rheumatoid factor; SAD: single ascending dose; SC: subcutaneous; SJC: swollen joint count; t_1/2_: terminal half-life; TNF-α: tumor necrosis factor α; TJC: tender joint count.

## Competing interests

BE, DL, CO, JG, MW, MT, JX, JL and JD are or were employees of Genentech during the study. BR declares an advisory relationship with Genentech. Genentech funded the study and oversaw the collection of data, but the data analysis and interpretation were performed with the assistance of expert advisors.

## Authors' contributions

BE served as a medical monitor, performed data analysis and drafted the manuscript. DL was responsible for data management, performed data analysis and drafted the manuscript. CO participated in trial design and execution and reviewed the manuscript. JG generated the molecule under study, served as scientific advisor and drafted the manuscript. MW generated the PD data, performed data analysis and drafted the manuscript. MT performed PK/ATA data analysis and reviewed the manuscript. JX performed PK/ATA data analysis and drafted the manuscript. JL participated in trial design, served as a medical monitor, performed data analysis and reviewed the manuscript. JD participated in trial design, performed data analysis and drafted the manuscript. BR was the principal investigator, performed data review and reviewed the manuscript. All authors read and approved the final manuscript.

## Supplementary Material

Additional file 1 **Maximum decreases in CXCL13 (pharmacodynamic effects) were consistently observed at serum pateclizumabconcentrations > 1 to 10 μg/ml across all cohorts**. The pateclizumab (circles) and CXCL13 (triangles) concentrations are presented as means ± SD. Click here for file

## References

[B1] Sfikakis PP The first decade of biologic TNF antagonists in clinical practice: lessons learned, unresolved issues and future directions Curr Dir Autoimmun 2010 11 180 210 20173395 10.1159/000289205

[B2] Bongartz T Sutton AJ Sweeting MJ Buchan I Matteson EL Montori V Anti-TNF antibody therapy in rheumatoid arthritis and the risk of serious infections and malignancies: systematic review and meta-analysis of rare harmful effects in randomized controlled trials JAMA 2006 295 2275 2285 10.1001/jama.295.19.2275 16705109

[B3] Edwards JC Szczepanski L Szechinski J Filipowicz-Sosnowska A Emery P Close DR Stevens RM Shaw T Efficacy of B-cell-targeted therapy with rituximab in patients with rheumatoid arthritis N Engl J Med 2004 350 2572 2581 10.1056/NEJMoa032534 15201414

[B4] Gramaglia I Mauri DN Miner KT Ware CF Croft M Lymphotoxin αβ is expressed on recently activated naive and Th1-like CD4 cells but is down-regulated by IL-4 during Th2 differentiation J Immunol 1999 162 1333 1338 9973387

[B5] Ware CF Network communications: lymphotoxins, LIGHT, and TNF Annu Rev Immunol 2005 23 787 819 10.1146/annurev.immunol.23.021704.115719 15771586

[B6] Chiang EY Kolumam GA Yu X Francesco M Ivelja S Peng I Gribling P Shu J Lee WP Refino CJ Balazs M Paler-Martinez A Nguyen A Young J Barck KH Carano RA Ferrando R Diehl L Chatterjea D Grogan JL Targeted depletion of lymphotoxin-α-expressing TH1 and TH17 cells inhibits autoimmune disease Nat Med 2009 15 766 773 10.1038/nm.1984 19561618

[B7] Browning JL Inhibition of the lymphotoxin pathway as a therapy for autoimmune disease Immunol Rev 2008 223 202 220 10.1111/j.1600-065X.2008.00633.x 18613838

[B8] Timmer TC Baltus B Vondenhoff M Huizinga TW Tak PP Verweij CL Mebius RE van der Pouw Kraan TC Inflammation and ectopic lymphoid structures in rheumatoid arthritis synovial tissues dissected by genomics technology: identification of the interleukin-7 signaling pathway in tissues with lymphoid neogenesis Arthritis Rheum 2007 56 2492 2502 10.1002/art.22748 17665400

[B9] Weyand CM Seyler TM Goronzy JJ B cells in rheumatoid synovitis Arthritis Res Ther 2005 7 Suppl 3 S9 S12 10.1186/ar1737 15960820 PMC2833971

[B10] Young J Yu X Wolslegel K Nguyen A Kung C Chiang E Kolumam G Wei N Wong WL DeForge L Townsend MJ Grogan JL Lymphotoxin-αβ heterotrimers are cleaved by metalloproteinases and contribute to synovitis in rheumatoid arthritis Cytokine 2010 51 78 86 10.1016/j.cyto.2010.03.003 20356761

[B11] Takemura S Braun A Crowson C Kurtin PJ Cofield RH O'Fallon WM Goronzy JJ Weyand CM Lymphoid neogenesis in rheumatoid synovitis J Immunol 2001 167 1072 1080 11441118 10.4049/jimmunol.167.2.1072

[B12] Gommerman JL Browning JL Lymphotoxin/light, lymphoid microenvironments and autoimmune disease Nat Rev Immunol 2003 3 642 655 10.1038/nri1151 12974479

[B13] Weyand CM Immunopathologic aspects of rheumatoid arthritis: who is the conductor and who plays the immunologic instrument? J Rheumatol Suppl 2007 79 9 14 17611973

[B14] Arnett FC Edworthy SM Bloch DA McShane DJ Fries JF Cooper NS Healey LA Kaplan SR Liang MH Luthra HS Medsger TA Jr Mitchell DM Neustadt DH Pinals RS Schaller JG Sharp JT Wilder RL Hunder GG The American Rheumatism Association 1987 revised criteria for the classification of rheumatoid arthritis Arthritis Rheum 1988 31 315 324 10.1002/art.1780310302 3358796

[B15] Wang H Cain G Kaiser R McBride J Lutman J Grogan J Gelzleichter T Iyer S Evaluation of a humanized monoclonal antibody targeting lymphotoxin a in non-human primates Ann Rheum Dis 2010 69 181 10.1136/ard.2008.101857 19176545

[B16] Shi K Hayashida K Kaneko M Hashimoto J Tomita T Lipsky PE Yoshikawa H Ochi T Lymphoid chemokine B cell-attracting chemokine-1 (CXCL13) is expressed in germinal center of ectopic lymphoid follicles within the synovium of chronic arthritis patients J Immunol 2001 166 650 655 11123349 10.4049/jimmunol.166.1.650

[B17] Rosengren S Wei N Kalunian KC Kavanaugh A Boyle DL CXCL13: a novel biomarker of B-cell return following rituximab treatment and synovitis in patients with rheumatoid arthritis Rheumatology (Oxford) 2011 50 603 610 10.1093/rheumatology/keq337 21098574

[B18] Rioja I Hughes FJ Sharp CH Warnock LC Montgomery DS Akil M Wilson AG Binks MH Dickson MC Potential novel biomarkers of disease activity in rheumatoid arthritis patients: CXCL13, CCL23, transforming growth factor α, tumor necrosis factor receptor superfamily member 9, and macrophage colony-stimulating factor Arthritis Rheum 2008 58 2257 2267 10.1002/art.23667 18668547

[B19] Meeuwisse CM van der Linden MP Rullmann TA Allaart CF Nelissen R Huizinga TW Garritsen A Toes RE van Schaik R van der Helm-van Mil AH Identification of CXCL13 as a marker for rheumatoid arthritis outcome using an in silico model of the rheumatic joint Arthritis Rheum 2011 63 1265 1273 10.1002/art.30273 21305530

[B20] Genovese MC Greenwald MW Alloway JA Baldassare AR Chase W Newman C Weaver ML Efficacy and safety of Baminercept in the treatment of rheumatoid arthritis (RA): results of the phase 2B study in the TNF-IR population [abstract] Arthritis Rheum 2009 60 Suppl 10 417

[B21] Isaacs JD Genovese MC Emery P Scheinberg MA Spindler AJ Newman C Weaver ML Efficacy and safety of Baminercept in the treatment of rheumatoid arthritis (RA): preliminary results of the phase 2B study in the DMARD-IR population [abstract] Arthritis Rheum 2009 60 Suppl 10 416

[B22] Grogan JL Wallach D, Kovalenko A, Feldmann M Novel mechanism of action for anti-lymphotoxin-α in autoimmune disease: depletion of Th1 and Th17 cells [abstract] Advances in TNF Family Research (Advances in Experimental Medicine and Biology), Proceedings of the 12th International TNF Conference, 2009 2011 New York: Springer 723

