# Supplementary material for: Safety, pharmacokinetics, and biologic activity of pateclizumab, a novel monoclonal antibody targeting lymphotoxin α: results of a phase I randomized, placebo-controlled trial
Source: Arthritis Res Ther. 2012 Jan 8;14(1):R6. doi: 10.1186/ar3554 (PMC3392792; doi:10.1186/ar3554)
Supplement: Supplementary file 1 [file ar3554-S1.DOC]

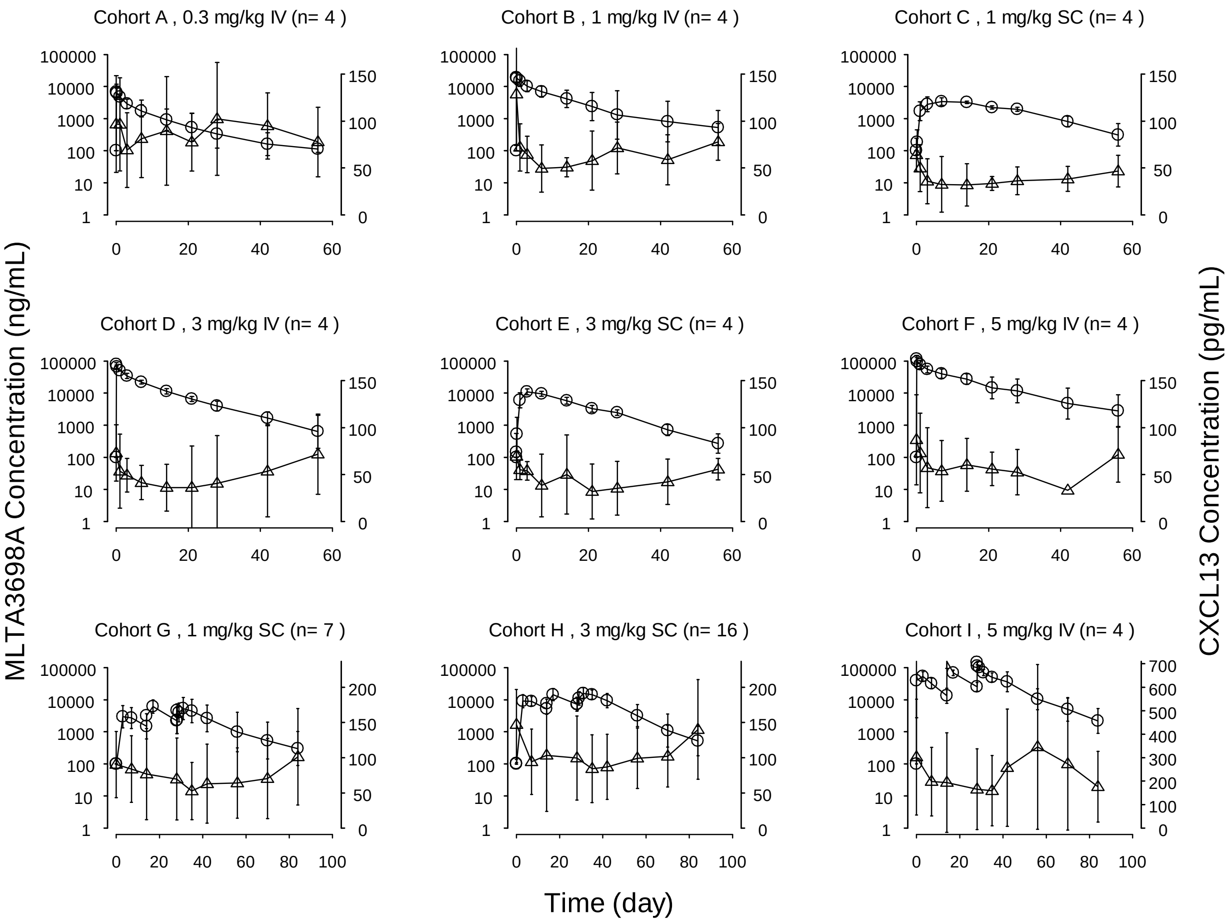
Cohort A , 0.3 mg/kg IV (n= 4)
Cohort B , 1 mg/kg IV (n= 4)
Cohort C , 1 mg/kg SC (n= 4)
Cohort D , 3 mg/kg IV (n= 4)
Cohort E , 3 mg/kg SC (n= 4)
Cohort F , 5 mg/kg IV (n= 4)
Cohort G , 1 mg/kg SC (n= 7)
Cohort H , 3 mg/kg SC (n= 16)
Cohort I , 5 mg/kg IV (n= 4)
MLTA3698A Concentration (ng/mL)
CXCL13 Concentration (pg/mL)
Time (day)
